# Impact of High-Shear Homogenization Pretreatment on Process Productivity, Economic Feasibility, and Product Quality During Long-Term Crossflow Microfiltration of Andean Blackberry Juice

**DOI:** 10.3390/foods15142493

**Published:** 2026-07-14

**Authors:** Pablo Rodríguez, Juan Zuluaga, Santiago González, Victoria Escobar, Misael Cortés, Fabrice Vaillant

**Affiliations:** 1 Corporación Colombiana de Investigación Agropecuaria-Agrosavia, Centro de Investigación La Selva, km. 7, vía Rionegro—Las Palmas, Sector Llanogrande, Rionegro-Antioquia, Research Unit ITAV: Innovaciones Tecnológicas para agregar Valor a Recursos Agrícolas, Rionegro 054048, Colombia; sgonzalezp@agrosavia.co (S.G.); jovescobarca@unal.edu.co (V.E.); fvaillant@agrosavia.co (F.V.); 2 Departamento Ingeniería Agrícola y Alimentos, Facultad Ciencias Agrarias, Universidad Nacional de Colombia sede Medellín, Medellín 050012, Colombia; jdzuluagan@unal.edu.co (J.Z.); mcortesro@unal.edu.co (M.C.); 3 French Agricultural Research Centre for International Development (CIRAD), UMR Qualisud, Rionegro 054048, Colombia; 4 Joint Research Unit—UMR Qualisud, Univ Montpellier, Avignon Universite, CIRAD, Institut Agro, IRD, Universite de La Reunion, 34000 Montpellier, France

**Keywords:** Andean blackberry juice, non-thermal juice processing, crossflow microfiltration, high-shear homogenization, flux decline kinetics, regime analysis, techno-economic feasibility

## Abstract

Although CFM is a promising non-thermal stabilization technology for blackberry juice, its industrial application is limited by permeate flux decline during long-term operation, while most previous studies have focused on short processing times. This study evaluated the effect of high-shear homogenization prior to enzymatic depectination on flux decline, product quality, and techno-economic feasibility during CFM. Juice processed by conventional grinding, high-shear homogenization, and enzymatic treatment was filtered through a 0.2-µm ceramic membrane at 150 kPa using feed volumes of 100–400 L. Homogenization reduced particle size and suspended insoluble solids, resulting in higher permeate flux, improved flux stability, and greater productivity. Flux decline analysis showed that high-shear homogenization extended the stable filtration regime and delayed severe fouling, sustaining an average *J_px_* of 65.3 L h^−1^ m^−2^ at VCR ~30 with feed volumes up to 400 L. Product quality was preserved, ensuring microbial reduction while improving anthocyanin and ellagitannin recovery (95% and 80%, respectively) and enhancing blackberry aroma. In addition, HS3+E reduced energy consumption and beverage production cost while achieving a positive NPV and a 21% IRR. Overall, homogenization improved the industrial feasibility of long-term CFM processing of Andean blackberry juice.

## 1. Introduction

Andean blackberry (*Rubus glaucus* Benth.) is an important perennial fruit cultivated across the Andean highlands of Colombia and Ecuador, where it is predominantly grown by smallholder farmers on plots often smaller than 1 ha, contributing significantly to rural employment and household income while supporting local fresh and processing markets. However, this fruit is highly perishable, exhibiting rapid softening and susceptibility to microbial spoilage shortly after harvest, with fresh fruits maintaining acceptable quality for only a few days under ambient conditions and up to approximately 8 days under refrigeration before quality declines due to firmness loss and microbial growth [1].

Despite these challenges, Andean blackberry is recognized for its rich phytochemical profile, including high levels of anthocyanins, ellagitannins, and other phenolic compounds associated with antioxidant, anti-inflammatory activity and other potential health benefits. The combination of short postharvest life and high functional value underscores the need for effective gentle processing technologies to reduce postharvest losses and preserve phytochemical quality while enhancing market opportunities for small-scale growers [2].

Thermal pasteurization (LTLT or, more commonly used for juices, HTST) is widely used to ensure microbial safety and extend the shelf life of fruits. However, the literature consistently shows a quality trade-off: heating accelerates degradation of thermolabile bioactive compounds (BACs), particularly anthocyanins, through mechanisms that lead to pigment loss, browning, and changes in the overall phenolic profile. These changes can reduce antioxidant capacity and modify the characteristic “fresh fruit” sensory taste and flavor of berry juices [3]. Studies in dark-colored berry matrices (e.g., blackberry) report measurable decreases in anthocyanins after pasteurization, while some phenolic families can be more stable depending on time–temperature intensity (e.g., ellagitannins sometimes showing higher retention than anthocyanins) [4]. These compositional shifts are frequently associated with sensory impacts, mainly via color changes and aroma/taste differences, which are key drivers of consumer perception [5]. From an investment and operating-cost perspective, pasteurization (HTST) remains advantageous at a large industrial scale; however, its energy demand and total cost of ownership depend strongly on heat-exchange design, pumping requirements, and heat recovery/regeneration efficiency [6].

These factors can become critical constraints for the adoption of this process in small and medium agroenterprises, where access to capital investment, maintenance capacity, and energy resources is often limited. Although CFM has demonstrated clear advantages as a mild stabilization technology for fruit juices [7,8], and recent advances such as flux optimization through backpulsing have been reported, these studies have been restricted to short-term operations (≈1 h) and low volume reduction factors (VCR ≈ 11) [9]. In preliminary trials, the application of repeated backpulse cycles under extended operating conditions resulted in reduced membrane operational stability, suggesting that this strategy may not be suitable for prolonged industrial processing. Furthermore, while integrated ultra-clean packaging systems have been proposed [10], the long-term industrial performance of CFM for Andean blackberry juice remains largely unexplored, representing a critical knowledge gap. Under extended operating conditions typical of industrial work shifts, key limiting phenomena such as membrane fouling and cake layer formation may be exacerbated, leading to significant flux decline and potentially compromising process viability. In this context, the novelty of this work lies in evaluating CFM performance under prolonged, industry-relevant conditions. Although it has been reported that particle size distribution and suspended solids significantly influence CFM productivity and fouling behavior [11], there are no studies evaluating these effects under long-term operational conditions representative of industrial processing. Therefore, the objective of this study was to assess the effect of homogenization as a physical pretreatment prior to microfiltration on process productivity, retention of bioactive compounds, sensory attributes, and economic feasibility during the crossflow microfiltration of Andean blackberry juice under extended operating conditions representative of industrial-scale production.

## 2. Materials and Methods

### 2.1. Raw Material

Andean blackberry (*Rubus glaucus* Benth.) fruits were harvested at commercial maturity stages 5 and 6 (maturity index ranging from 2.6 ± 0.2 to 4.8 ± 0.2, according to NTC 4106). The fruits were supplied by smallholder farmers’ association ASOFRUTAS (La Ceja, Antioquia, Colombia). The study was carried out at the agro-industrial pilot plant of the La Selva Research Center (Rionegro, Antioquia, Colombia).

### 2.2. Andean Blackberry CFM Process

As an initial approach, a backpulsing strategy was evaluated following the methodology reported by Zuluaga et al. [9]; however, this condition led to membrane damage under the operating conditions tested. Therefore, subsequent experiments did not include backpulsing as part of the CFM process configuration. Instead, the study focused on physical pretreatments aimed at reducing particle size. In this context, homogenization was selected as the main pre-treatment strategy. Also, a preliminary study was conducted to assess whether pre-treatment affected the sensory quality of the juice.

**Figure A1:**
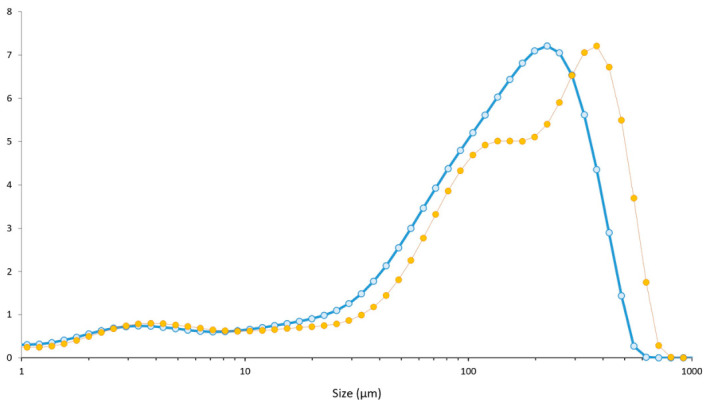
Particle-size distribution obtained during the preliminary evaluation of particle-size reduction systems for the CFM process. The yellow curve represents the high-shear homogenizer (HS3), and the blue curve represents the colloidal mill.

Two particle size reduction systems were evaluated to reduce the particle size of whole Andean blackberry fruits during 7 min of treatment: a rotor–stator colloidal mill (7-min trial) (CM-60, Centricol, Medellín, Colombia) and a rotor-stator high-shear homogenization tri-emulsifying pump (TRL-L3-60, Wenzhou Rayen Machinery Co., Ltd., Wenzhou, China), powered by a 4.0-kW Siemens motor (Siemens AG, Munich, Germany) operating at 2900 rpm, referred to as HS3. Particle-size distributions obtained with both systems are presented in Appendix A, Figure A1. Although the colloid mill achieved a moderate reduction in particle size, it generated greater seed disruption, whereas intact seeds were generally preserved after HS3 processing. This difference was associated with increased bitterness and astringency in the colloidal mill-treated mash. A preliminary sensory evaluation conducted by a trained panel (n = 18) indicated that blackberry mash produced with the colloidal mill exhibited substantially higher bitterness and astringency (approximately 7 on a 10-point intensity scale; 0 = not perceived, 10 = extremely intense) than mash produced using the GR or HS3 treatments (approximately 3). Because lower astringency was associated with superior overall product quality and consumer acceptability, the colloidal mill was not selected for further experimentation, and all subsequent trials were performed using the HS3 system.

**Figure 1:**
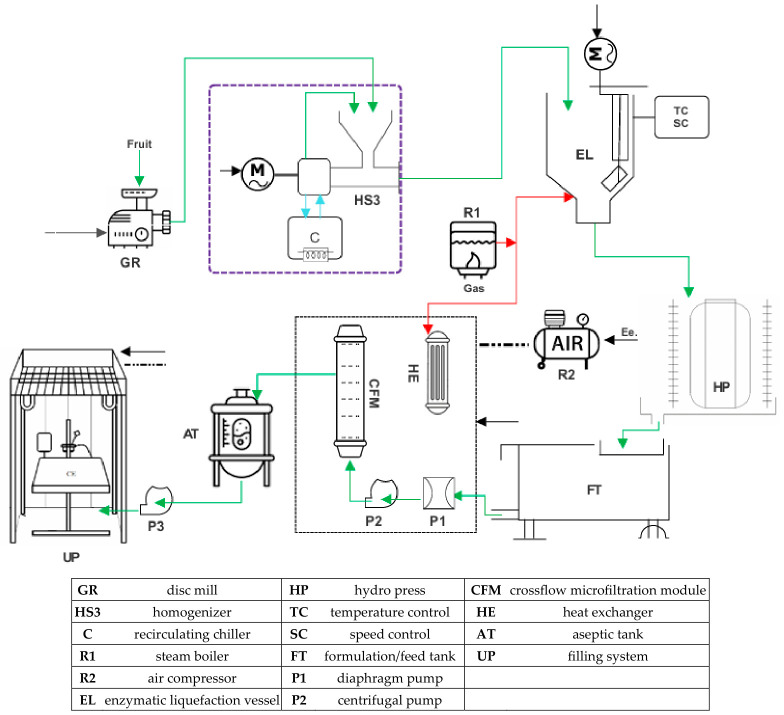
Integrated CFM process line to produce Andean blackberry juice. Line conventions: green lines indicate product flow; red lines indicate steam supply; blue lines indicate water circulation; black lines indicate electrical energy; and dashed lines indicate compressed air.

To evaluate the impact of homogenization on microfiltration performance, two feed preparation strategies were implemented. In the first treatment, called as unit operation Grinder (GR, see Figure 1), fruits were mechanically milled using a disc mill (Javar M32I, Javar, Bogotá, Colombia) equipped with a 4.5-mm gap opening. In the second treatment, the milled pulp was subjected to high-shear homogenization using a HS3. Temperature during homogenization was controlled below 30 °C using a 20-L recirculating chiller (Centricol, Medellín, Colombia) (see the purple dotted line, Figure 1). Homogenization was performed at a pulp-to-water mass ratio of 7:3 with a recirculation time of 7 min.

Following size reduction, the mixture underwent enzymatic depectinization in a double-jacketed vessel with temperature control (GAME Co., Medellín, Colombia) at 35 °C for 1 h under continuous agitation (see Figure 1). A commercial multi-enzyme preparation (Pectinex^®^ Ultra SP-L; Novonesis A/S, Bagsværd, Denmark), containing pectinases, hemicellulases, and β-glucanases derived from *Aspergillus aculeatus* (declared activity: 3300 PGNU·g^−1^) was added at 150 ppm (*w*/*w*).

The enzymatically treated mash was then pressed (HP, Figure 1) using a vertical hydraulic press (300 L; Enotecnica Pillan, Vicenza, Italy) fitted with a 1.2-mm external mash and an expandable internal membrane, applying a maximum pressure of 300 kPa for juice extraction. The resulting press cake was washed and repressed in two additional cycles to maximize the recovery of soluble bioactive compounds from drupe tissues and residual solids.

To obtain formulated raw juice with 40% fruit content, water was added to a 400-L feed tank (FT, Figure 1) at a volume equivalent to twice the initial mass of the raw material. Total soluble solids (TSSs) were adjusted to 10.5 ° Brix by adding approximately 7% (*w*/*w*) sugar.

The CFM unit (gray dotted square, see Figure 1) was locally built (GAME Co., Medellín, Colombia) and consisted of a double-diaphragm pump (P1, Figure 1) (SaniForce^®^ 515) used for pressurized raw juice feed supply, and a centrifugal pump (P2, Figure 1) (QIS series, 7.5 HP; 50/60 Hz) used to generate a tangential flow velocity of 6 m·s^−1^. Microfiltration was performed using a module equipped with three tubular ceramic membranes (CFM, Figure 1) (α-Al_2_O_3_, Membralox GP-IC EP4840, Bazet, France). Each membrane contained 48 channels with an internal diameter of 4.0 mm and a nominal pore size of 0.2 μm. The membranes, each 1020 mm in length, were vertically installed within the module, providing a total effective filtration area of 1.8 m^2^ and sealed with elastomeric EPDM gaskets. The permeate flow rate was continuously monitored using an electromagnetic flowmeter (Sitrans f m mag 5000, Siemens).

All experiments were conducted in continuous feed mode without retentate withdrawal. System temperature was maintained at 35 °C using a shell-and-tube heat exchanger (HE, Figure 1). Feed pressure (Pf) and permeate pressure (Pp) were measured using diaphragm pressure gauges to maintain a constant trans-membrane pressure (TMP) of 150 kPa [9]. Flow circulation and operational parameters were controlled via solenoid valves connected to a programmable logic controller (PLC Xinje TG765-UT, Xinje Electronic Co., Ltd., Wuxi, China) and operated using an HMI touchscreen interface. The permeate was collected in an aseptic tank (AT, Figure 1) coupled to an ultra-clean packaging system (UP, Figure 1) consisting of a laminar airflow cabinet equipped with a HEPA 0.3-um filter and a semi-manual filling machine (Sympaty TOP 320^®^, Villefranche-sur-Saône, France) with an AL40 side-channel pump (Tellarini & C., Lugo, Italy) (P3, Figure 1). The Andean blackberry micro filtrated juice was immediately packed into pre-irradiated multilayer bags (LDPE/metallized PET/LDPE; FLEXBAG, Santa Anita, Lima, Peru) equipped with V-pull valves.

#### Membrane Cleaning in Place (CIP) Procedure

The CIP sequence consisted of an initial rinse with neutral water, followed by two alkaline cleaning cycles using a 1.5% (*w*/*w*) NaOH solution at 90 °C for 20 min. Subsequently, an acidic treatment was performed using a 1% (*w*/*w*) HNO_3_ solution at 90 °C for 20 min. The protocol concluded with a final neutralization rinse. The cleaning procedure was carried out according to established membrane cleaning guidelines [12]. The permeate piping and aseptic tank were sterilized with steam at 210 kPa during 20 min, while pressure was regulated using a vent filter (PES, 0.2 µm).

### 2.3. Process Productivity

#### 2.3.1. Permeate Flux (*J_px_*)

The permeate flux (*J_px_*) was determined from the volume of permeate collected (V_p_, L), the total membrane filtration area (A, m^2^), and the effective operating time (t, h), according to Vaillant et al. [13] as expressed in Equation (1):

(1) $$J_{px}=\frac{V_{p}}{A*t}$$

To fit the flowmeter data to the experimental work volumes (instant flux), to avoid overestimated values, the *J_px_* values were trending by the gel-polarization model, a mechanistic model often used for the adjustment of experimental data as a function of the logarithm of VCR according to Zuluaga et al. [9], as expressed in Equation (2):

(2) $${Jp}_{inst}\;=\;Kc\;Ln\;\left(\frac{cg}{cb}\right)$$

where *K*c represents the overall mass transfer coefficient; (cg/cb) is the ratio between the concentration of retained material in the bulk of retentate (cb) and the concentration at the membrane interface (cg); cg is a constant, characteristic for a given fooling layer (cg is the final concentration when *J_px_* is zero). Subsequently, the cumulative permeate volume as a function of time was estimated for each treatment.

#### 2.3.2. Volume Reduction Factor (VCR)

The VCR, which indicates the degree of feed concentration resulting from permeate extraction through the membrane, was calculated according to Equation (2), where Vf corresponds to the initial feed volume and Vr to the final retentate volume [14].

(3) $$VCR=\frac{V_{f}}{V_{r}}=1+(\frac{V_{p}}{V_{r}})$$

#### 2.3.3. Flux Decline Analysis

The decline *J_pinst_* was used to evaluate the impact of physical pretreatments on permeate flux. The first step consisted of comparing the GR+E and HS3+E treatments using 200 L of formulated raw juice as the feed volume.

##### Mathematical Procedure

To quantitatively identify regime transitions and characterize *J_pinst_* decline kinetics, the following procedure was applied:

(a) Apparent decay coefficient

The instantaneous apparent *J_pinst_* decline coefficient (λ_i_) was calculated as follows:

(4) $$\lambda_{i}=-\frac{ln(J_{i})-ln(J_{i-1})}{t_{i}-t_{i-1}}$$

where *J_i_* and *J_i−_*_1_ are the flux values at consecutive time points *t_i_* and *t_i−_*_1_, respectively.

(b) Smoothing

To reduce noise and facilitate trend detection, the $\lambda_{i}$ series was smoothed using a five-point moving average, as expressed in Equation (5):

(5) $${\bar{\lambda }}_{i}=\frac{1}{5}\sum_{k=i-2}^{i+2}\lambda_{k}$$

where ${\bar{\lambda }}_{i}$ is the smoothed apparent decay coefficient.

(c) Segmentation and breakpoint detection

The natural logarithm of the smoothed apparent decay coefficient, $ln(\bar{\lambda })$, was used to identify regime transitions. Two transition times ($t_{12}$ and $t_{23}$) were determined by minimizing the total sum of squared errors (SSEs) across three consecutive regression segments:

(6) $$\begin{array}{c} SSE_{Total}=SSE_{1}+SSE_{2}+SSE_{3} \end{array}$$

where

(7) $$\begin{array}{c} SSE_{j}=\sum_{i=1}^{n_{j}}(y_{i}-{\hat{y}}_{i}{)}^{2} \end{array}$$

and $y_{i}$ and ${\hat{y}}_{i}$ represent the observed and predicted values of $ln(\bar{\lambda })$, respectively.

The optimal breakpoint locations corresponded to the combination of $t_{12}$ and $t_{23}$ that minimized $SSE_{Total}$.

(d) Model fitting by regime

Based on the identified temporal boundaries, flux decline was modeled independently in each regime: based on the identified breakpoints, the filtration process was divided into three temporal regions:

Regime I: 0 < t ≤ t_12_

Regime II: t_12_ < t ≤ t_23_

Regime III: t > t _23_

Flux decline was modeled independently in each region using a piecewise formulation:

$$j_{\left(t\right)}=\;\begin{array}{cc} A\;e^{{-k}_{1}t}, & 0<t\leq t_{12}\\ a_{2}+\;b_{2}t, & t_{12}<t\leq t_{23}\\ a_{3}+\;b_{3}t, & t_{23} \end{array}$$

where *A* and *K*_1_ are the exponential model parameters for Region I while a_2_, b_2_ and a_3_, b_3_ are the linear regression coefficients for Regions II and III, respectively.

(e) Goodness-of-fit evaluation

The quality of fit for each regime was evaluated using the coefficient of determination ($R^{2}$):

(8) $$\begin{array}{c} R^{2}=1-\frac{\sum_{i=1}^{n}(J_{i}-{\hat{J}}_{i}{)}^{2}}{\sum_{i=1}^{n}(J_{i}-\bar{J}{)}^{2}} \end{array}$$

and the root means square error (RMSE):

(9) $$\begin{array}{c} RMSE=\sqrt{\frac{1}{n}\sum_{i=1}^{n}(J_{i}-{\hat{J}}_{i}{)}^{2}} \end{array}$$

where $J_{i}$, ${\hat{J}}_{i}$, and $\bar{J}$ are the observed, predicted, and mean experimental flux values, respectively.

##### Software Implementation

Data processing, including calculation of the apparent decay coefficient (λ*i*), moving-average smoothing, breakpoint detection, segmented regression, model fitting, and goodness-of-fit evaluation, was performed using MATLAB R2023a (MathWorks, Natick, MA, USA). The complete computational workflow is provided as Supplementary Code S1 and excel data set to facilitate reproducibility and application to independent datasets.

Uncertainty associated with the estimated transition times (t_1-2) and (t_2-3) was evaluated using bootstrap resampling (1000 iterations), and the corresponding 95% confidence intervals were calculated from the bootstrap distributions. Model parameters for each regime are reported together with the fitted equations. Model adequacy was assessed using the coefficient of determination (R^2^) and the root mean square error (RMSE).

The productivity was calculated as follows:

(10) $$AUC=\;\int_{t_{i}}^{t_{f}}j\left(t\right)dt$$

The numerical analysis was done using the trapeze rule:

(11) $${AUC}_{i}=\;\frac{j_{i}+\;j_{i-1}}{2}\;\left(t_{i\;}-\;t_{i-1\;}\right)$$

The Hermia models [15] (time-based forms) for the fouling mechanisms were applied.
**Mechanism****Linearized Form****Plot**Complete blockingln J = ln J_0_ − *k*tln J vs. tIntermediate blocking1/J = 1/J_0_ + *k*t1/J vs. tStandard blocking1/√J = 1/√ J_0_ + *kt*1/√J vs. (t)Cake filtration1/J^2^ = 1/J_0_^2^ + *k*t1/J vs. tThe R^2^ was estimated after the linearization of J and t.

For the HS3+E CFM process for a long period, the same decline was applied for a feed volume of 300 and 400 L to consider the CIP time within an industrial workday (8 h). The same calculation procedure used to determine flux decline was consistently applied.

### 2.4. Particle Size Distribution

To evaluate the effect of GR and HS3 pretreatments before and after the enzymatic maceration on feed microstructure, the hydro pressed juice was characterized in terms of particle size distribution. Measurements were performed by laser diffraction using a mastersizer 3000 analyzer (Malvern Instruments Ltd., Worcestershire, UK) equipped with a Hydro LV dispersion unit, following the protocol described by Dahdouh et al. [16]. The optical parameters used were a refractive index of 1.45 for the dispersed phase (CSCG), 1.33 for water, an absorption index of 0.5, and an obscuration level of 11.24%.

### 2.5. Quality Analysis

#### 2.5.1. Physicochemical Analyses

Suspended insoluble solids (SISs) were quantified following the methodology described by Vaillant et al. [8]. Briefly, 15 g of sample were centrifuged at 5000 rpm for 10 min using a refrigerated centrifuge (SL16R, Thermo Scientific, Waltham, MA, USA). The supernatant was carefully decanted and drained, and the remaining solid residue was considered the SIS fraction. All analyses were performed in triplicate.

Method repeatability was evaluated from the relative standard deviation (RSD) of all SIS determinations conducted throughout the study. The average RSD was 2.9%, with values ranging from 0.46% to 5.93%, indicating good analytical repeatability. The centrifugation conditions were selected based on methodologies previously applied for the determination of suspended insoluble solids in fruit juices during crossflow microfiltration studies, particularly those reported by Vaillant et al. [8], for blackberry juice clarification.

#### 2.5.2. Microbiological Analysis

Microbiological analyses were performed on the raw juice and permeate fractions corresponding to the clarified ready-to-drink Andean blackberry juice, following AOAC methods with minor modifications. Aerobic mesophilic bacteria [17], total and fecal coliforms according to development method by turner et al. [18], mesophilic lactic acid bacteria [19], yeasts and molds [20]. All analyses were conducted in triplicate, and laboratory procedures were carried out in accordance with ISO standards [21]. The microbiological results were expressed as decimal logarithms of colony-forming units per milliliter (log CFU/mL), following standard recommendations for the handling and proper interpretation of microbiological data [22]. Microbiological results were expressed as decimal logarithms of colony-forming units per milliliter (log CFU/mL). The limit of detection (LOD) for plate-count methods was 1.0 log CFU/mL, corresponding to 10 CFU/mL in the analyzed sample. Counts below the LOD were reported as <1.0 log CFU/mL. Microbial reduction was calculated as the difference between the microbial load of the raw juice and that of the corresponding permeate fraction (log reduction = log N0 − log Nt), where N0 and Nt represent the microbial counts before and after microfiltration, respectively.

#### 2.5.3. Sensory Analysis

The sensory properties of the samples were characterized through Quantitative Descriptive Analysis (QDA) [23]. Eighteen semi-trained assessors (25–58 years of age) from the La Selva Research Center (Agrosavia, Colombia) participated in the evaluation. Prior to the sensory evaluation, panelists underwent a structured training program based on ISO standards [24]. The training consisted of four stages: (i) recruitment and preliminary selection, (ii) theoretical–practical training, (iii) panelist selection, and (iv) periodic recalibration sessions. During training, panelists were familiarized with the sensory descriptors and intensity scaling procedures. Reference solutions were used to identify and calibrate basic tastes, including sucrose (sweet), citric acid (sour), caffeine (bitter), sodium chloride (salty), and tannic acid (astringent). The blackberry aroma and flavor descriptors were trained using healthy, fully mature Andean blackberry fruits. A sensory lexicon was developed by consensus among the trained assessors. The final descriptors used for QDA were blackberry aroma, fermented aroma, blackberry flavor, sourness, sweetness, bitterness, fermented flavor, astringency, and overall quality. The intensity of each attribute was evaluated using an unstructured scale, and panel consensus was periodically verified throughout the study. Samples were served at 8–10 °C in randomized, three-digit coded 30 mL cups and evaluated in individual booths under controlled environmental conditions. Sensory assessment was performed in morning and afternoon sessions, considering odor, taste, sensory sensations, and overall quality. The intensity of each attribute was scored on a structured 10-point scale (0 = not perceived; 10 = extremely intense). To reduce potential carryover effects, assessors rinsed their mouths with water between evaluations.

#### 2.5.4. Bioactive Compounds Analysis

- Reagents

All reagents used for analytical determinations were of analytical grade. Solvents used for UHPLC analyses were of HPLC grade. Methanol (purity ≥ 99.8%) and acetonitrile (LC/MS grade) were purchased from Merck (Darmstadt, Germany), while formic acid was obtained from Sigma-Aldrich Chemie (Steinheim, Germany) and dimethyl sulfoxide (DMSO) from J.T. Baker. Ellagic acid and cyanidin-3-glucoside chloride (purity ≥ 98%) were supplied by PhytoLab GmbH & Co (Vestenbergsgreuth, Germany). Ultrapure water used in all analyses was produced using a Millipore Simplicity purification system.

**Figure A2:**
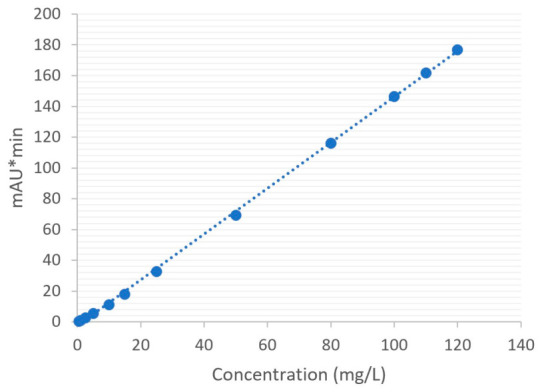
Calibration curve cyanidin 3-glucoside chloride (C3G) HPLC-PDA method. R^2^ = 0.9995. Equation: Y = 1.4859 X − 2.4808. The dotted line represents the linear regression fit.

**Figure A3:**
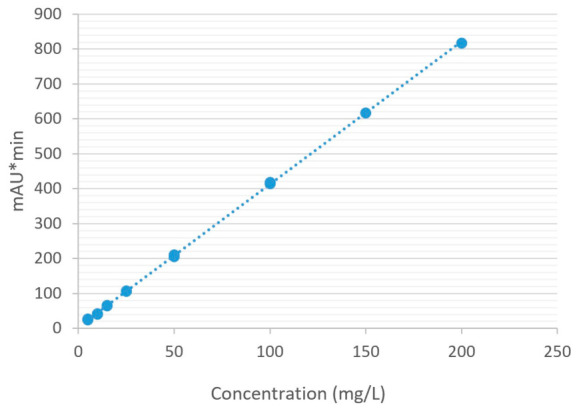
Calibration curve ellagic acid by the HPLC-PDA method. R^2^ = 0.9998. Equation: Y = 4.0897X + 3.7969. The dotted line represents the linear regression fit.

Anthocyanins and ellagitannins were extracted following the method of García-Villalba et al. [25] with minor modifications adapted to the different matrices analyzed here. For raw and microfiltered juices, 5 mL of sample were mixed with 5 mL of extraction solvent. For retentate samples, 5 mL of sample were extracted using two successive 10 mL solvent washes and adjusted to a final volume of 20 mL with methanol because of their higher suspended solids content. Quantification was performed using external calibration curves, and concentrations were calculated after applying the corresponding dilution factors. Method validation confirmed adequate linearity and reliability for the quantification of cyanidin-3-O-glucoside and ellagic acid within the analyzed concentration ranges (Appendix B, Figure A2 and Figure A3, Table A1 and Table A2). The extraction solvent consisted of 0.5% (*v*/*v*) HCl in Type I water, methanol, and dimethyl sulfoxide in a 20:40:40 (*v*/*v*/*v*) ratio. Mixtures were agitated for 35 min, followed by centrifugation at 3000 rpm for 10 min at 20 °C. The supernatants were filtered through 0.22 μm nylon syringe filters and stored at −30 °C until chromatographic analysis. Quantification of cyanidins and ellagitannins was performed according to Mertz et al. [26] with minor modifications using a Vanquish UHPLC system (Thermo Scientific) equipped with a photodiode array detector. Chromatographic separation was achieved on a Hypersil Gold C18 reverse-phase column (100 × 2.1 mm, 1.9 μm; Merck, Germany) coupled to a guard column (10 × 2.1 mm, 1.9 μm). The mobile phase consisted of solvent A (water/formic acid, 98:2 *v*/*v*) and solvent B (water/acetonitrile/formic acid, 80:18:2 *v*/*v*/*v*), at a flow rate of 0.2 mL·min^−1^ and a column temperature of 45 °C. The injection volume was 5 μL. The gradient program was as follows: 0 min, 5% B; 12 min, 15% B; 18 min, 30% B; 35 min, 25% B; 40 min, 5% B, followed by 5 min of re-equilibration. Detection wavelengths were set at 515 nm for cyanidins and 254 nm for ellagitannins. External calibration curves were constructed using cyanidin-3-glucoside (0.5–120 mg·L^−1^; R^2^ = 0.9995, Appendix B, Figure A2) and ellagic acid (5–300 mg·L^−1^; R^2^ = 0.9993, Appendix B, Figure A3). Cyanidin-3-rutinoside, lambertianin C, and sanguiin H-6 contents were expressed as equivalents of the corresponding external standards. All analyses were performed in triplicate.

- Compounds retention

The recovery or retention of compounds in the retentate was calculated as the percentage of the initial compound mass present in the feed that remained in the retentate after filtration using Equation (12) according to authors Carmona et al. [27].

(12) $$R_{R\;}(\%)=100\frac{C_{R\;}V_{R}}{C_{F}V_{F}}$$

where (C_F_) and (C_R_) are the compound concentrations (mg L^−1^) in the feed and retentate, respectively, and (V_F_) and (V_R_) are the corresponding feed and retentate volumes (L).

### 2.6. Economic Evaluation Criteria for Andean Blackberry Juice Production

#### 2.6.1. Energy Consumption

The electrical energy consumption of the equipment involved in the CFM processing line (see Figure 1) was estimated using Equation (13). Electricity consumption was estimated from the power ratings reported in the manufacturers’ technical datasheets for each processing unit described in Section 2.2:

(13) Ee=P∗t/η

where E is the electrical energy consumption, P is the power of the equipment (kW), t is the operating time (h), and η is motor efficiency.

Electricity costs were estimated using the regulated industrial electricity tariff published by Empresas Públicas de Medellín (EPM), the local utility provider for the enterprise location (Rionegro, Antioquia, Colombia). The industrial tariff corresponding to the study period (2026) was used as the reference unit cost and applied consistently across all equipment included in the techno-economic analysis. Specific energy consumption (kW/L) was calculated relative to the total beverage volume obtained during the processing operation over a work shift.

#### 2.6.2. Financial Indicators

For calculating the operating expenses (OPEXs), we worked with a case study focused on technology transfer to rural communities. As the CFM process line was designed for implementation in small-scale agro-industries, this study was carried out in collaboration with an association of small growers named ASOFRUTAS (La Ceja, Antioquia, Colombia). First, a diagnosis of the infrastructure and service requirements (water, gas, electrical energy, see Figure 1) was conducted to estimate the investment necessary to strengthen the association’s capacity for the optimal operation of the equipment.

In addition to the equipment investment, the analysis also considered the capital required to build the CFM unit and the other processing equipment, including auxiliary systems such as the boiler and compressor (see Figure 1), as well as the ultraclean packaging system, in order to estimate the total cost of producing blackberry juice in its final presentation. Moreover, the requirements for human resources, raw materials, packaging bags, productivity, and other operational factors were estimated for long-term process operation.

Raw material costs were estimated using historical price data provided by ASOFRUTAS, the fruit growers’ association that supplied the blackberry used in this study. Labor costs were estimated according to Colombian labor regulations, using the statutory minimum wage for 2026 as the reference value for operational personnel requirements in the processing facility. In addition to base salary, mandatory labor provisions, including social security contributions and employer charges, were incorporated into the estimation to better reflect the full employment cost. This approach ensures that the economic analysis represents realistic operating conditions in the Colombian context. The number and qualification level of personnel required for continuous fruit processing were defined according to the expected workload under real operating conditions for the Andean blackberry CFM process. These tasks included juice preparation, equipment supervision, cleaning, and basic process control activities.

The economic evaluation was performed using a discounted cash flow (DCF) model following established techno-economic assessment (TEA) methodologies by Pintarič et al. [28].

The discount rate was estimated as the after-tax cost of debt according to the following:

(14) discount rate=Kd (1.T)

where Kd is the annual interest rate and T is the corporate income tax rate.

The selling price of the final product was estimated using a cost-plus pricing approach with a 30% markup over the unit production cost.

Project revenues, cash flows, NPV, IRR, and payback period were subsequently calculated following Viana et al. [29] (the assumptions are summarized in Appendix C (Table A3).

##### Net Present Value (NPV) Analysis

The net present value (NPV) was calculated to evaluate the economic feasibility of the process, as defined by the following expression:

(15) $$NPV=I0+\sum_{\left\{t=1\right\}}^{\left\{n\right\}}\frac{\left\{{CF}_{t}\right\}}{\left\{{\left(1+r\right)}^{t}\right\}}$$

where I0 is the initial capital investment, CFt is the net cash flow at year t, r is the discount rate, and n is the project lifetime (years).

##### Internal Rate of Return (IRR)

The IRR is the discount rate that equates the present value of future cash flows to the initial investment, such that the NPV equals zero. Mathematically, project IRR is obtained by solving the following:

(16) $$IRR=-I0+\sum_{\left\{t=1\right\}}^{\left\{n\right\}}\frac{\left\{{CF}_{t}\right\}}{\left\{{\left(1+r\right)}^{t}\right\}}$$

where I0 is the initial investment, CFt is the net cash flow at year t, r is the internal rate of return, and n is the project lifetime.

##### Payback Period

The time required for cumulative cash flows to equal the initial investment.

##### EBITDA

Financial ratios were used to assess the comparative valuation of the project.

The EV/EBITDA ratio was calculated according to the following equation:

(17) $$EBIDTA=\frac{EV}{\left\{EBITDA\right\}}$$

where EV represents Enterprise Value and EBITDA corresponds to earnings before interest, taxes, depreciation, and amortization.

Similarly, the EV/Sales ratio was calculated as follows:

$$\frac{EV}{Sales}$$

where Sales represents total annual revenues.

### 2.7. Experimental Design

The study was designed to evaluate permeate flux behavior and process productivity under different feed physical preparation strategies and processing volumes. Two pretreatment conditions were assessed: GR+E and HS3+E. The GR+E treatment was evaluated at processing volumes of 100 and 200 L, whereas the HS3+E treatment was evaluated at 100, 200, 300 and 400 L to assess performance under extended operation conditions.

### 2.8. Statistical Analysis

Quality analysis was measured in triplicate, and results are reported as mean ± standard deviation (SD). Normality and homoscedasticity were verified with Shapiro–Wilk and Levene tests, respectively. For compound retention, a one-way ANOVA was conducted when significant differences were detected (*p* < 0.05), while Tukey’s HSD was used for pairwise comparisons. The sensory product characterization test was performed to identify discriminants with sensory descriptors according to the GR+E and HS3+E process treatment. For each descriptor, an ANOVA model was applied to check whether the scores given by the assessors differed significantly. All analyses were performed in XLSTAT (2023.1.4, Addinsoft, Paris, France).

## 3. Results and Discussion

### 3.1. Productivity

**Figure 2:**
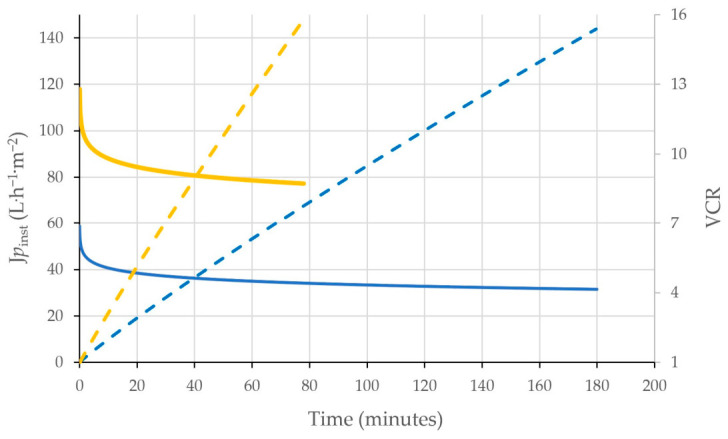
Flux decline dynamics of the GR+E and HS3+E treatments during crossflow microfiltration using a 200-L feed volume. Solid lines represent permeate flux (L·h^−1^·m^−2^), and dashed lines represent VCR. Blue lines correspond to GR+E treatment, while yellow lines correspond to HS3+E treatment. GR+E: screw-type grinder fitted with a 4.5-mm perforated plate. HS3+E: high-shear, three-stage rotor–stator inline homogenization treatment.

Figure 2 presents the temporal evolution of permeate flux for the GR+E and HS3+E treatments under crossflow microfiltration at the 200-L scale. In both cases, a characteristic flux decline pattern is observed, consisting of a rapid initial decrease followed by gradual stabilization over time, as has been published elsewhere and it is well known that membrane fouling is one of the main phenomena responsible for this [6,30]. Previously, Zuluaga et al. [9] reported higher flux (86 L·h^−1^·m^−2^) values for Andean blackberry juice under the same formulation (40% fruit content) and similar operating conditions (Pall microfiltration module, transmembrane pressure, and tangential velocity) working with a feed volume of 100 L and a VFR of 7.7. The difference observed in this study may be explained by the fact that approximately twice the amount of raw material and feed juice volume was processed compared with the previous work. This likely resulted in a higher concentration of SIS, which contributed to increased membrane fouling as will be discussed later. In this regard, Behnaz Razi et al. [31] indicated that suspended materials and cell debris accumulating on the membrane surface are responsible for permeate flux decline.

**Figure A4:**
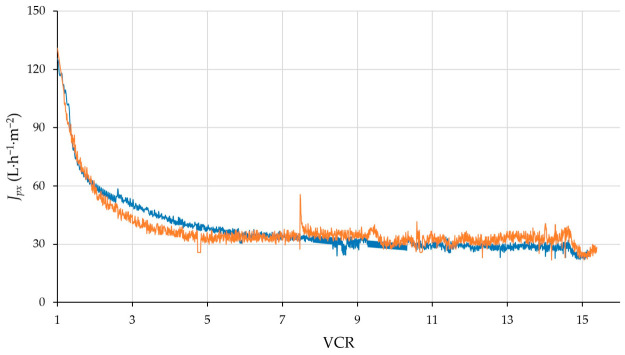
Flux behavior during the crossflow microfiltration process using a 200-L feed volume. GR+E treatment, blue line. Feed-and-bleed strategy, orange line.

These results indicate that increasing production volume requires the implementation of strategies to maintain or enhance permeate flux over time. Retentate extraction (feed and bleed) during crossflow microfiltration has been reported to improve performance by limiting the accumulation of suspended solids, pectins, and colloids, thereby reducing viscosity, concentration polarization, and cake layer formation, key factors responsible for flux decline in fruit juice filtration [32] Accordingly, a feed-and-bleed strategy was evaluated after the marked flux decline observed during the processing of 200 L of feeding volume; however, no significant improvement in permeate flux was observed (see Appendix D, Figure A4), suggesting that fouling was mainly governed by rapid cake formation and pore blocking rather than bulk concentration effects. Therefore, strategies based solely on controlling retentate concentration of insoluble solids may be insufficient to mitigate fouling. Instead, modifying the physicochemical characteristics of the feed, particularly particle size distribution and structural organization, becomes critical.

To further evaluate the impact of the HS3+E treatment, flux dynamics were analyzed by dividing the filtration process into distinct operational regions. The regime-segmented analysis is summarized in Table 1. Flux decline modeling parameters and performance metrics for GR+E and HS3+E treatments across filtration regimes further quantify these differences. The goodness-of-fit statistics (R^2^ and RMSE) for each fitted segment, together with the estimated transition times (t_12_ and t_23_) and their associated 95% confidence intervals, are reported in Appendix E, Table A4. Three distinct filtration regions were identified for both treatments, although their temporal boundaries differed. In regime 1, HS3+E showed a higher initial flux and a slower exponential decay rate compared to GR+E, suggesting reduced susceptibility to rapid pore blockage. In regime 2, both treatments exhibited a linear decline, but HS3+E maintained a higher average flux and a steeper slope, reflecting enhanced permeability under intermediate fouling conditions. In regime 3, corresponding to long-term operation, HS3+E continued to outperform GR+E, with higher final flux and cumulative productivity (AUC). Overall, the HS3+E treatment increased AUC by approximately 7.7%, 5.9%, and 9.6% in regimes 1, 2, and 3, respectively. In contrast, the average permeate flux increased by approximately 111%, 136%, and 158% across the three regimes, demonstrating that the effect of HS3+E was particularly pronounced in terms of flux enhancement and process productivity.

These observations are consistent with previous studies on crossflow microfiltration, which describe flux decline as a multi-stage process governed by evolving fouling mechanisms. Song et al. [33] reported that flux decline typically transitions from an initial non-equilibrium regime to a pseudo-steady-state condition, driven by the progressive development of fouling layers. Similarly, Hermia [34] and subsequent studies [35] have demonstrated that early filtration stages are dominated by pore blocking mechanisms, while long-term behavior is controlled by cake formation and concentration polarization. In food systems such as fruit juice filtration, these mechanisms are particularly relevant due to the presence of suspended solids, colloids, and macromolecules that contribute to both internal and surface fouling.

From a mechanistic perspective, the improved performance observed for HS3 can be attributed to modifications in fouling behavior across the three regimes. In regime 1, the reduced rate of flux decline suggests that HS3+E mitigates pore blocking, likely by altering particle size distribution, reducing the presence of fine particulates, or modifying physicochemical interactions between solutes and the membrane surface. In regime 2, the higher flux and sustained decline indicate a more permeable and less compact fouling layer, which may result from changes in particle adhesivity, water retention capacity, or reduced compressibility of the cake layer. Finally, in regime 3, the higher steady-state flux observed for HS3+E suggests a dynamic equilibrium between fouling deposition and shear-induced removal, characteristic of crossflow systems, but shifted toward lower hydraulic resistance.

Overall, the combination of higher initial flux, slower decline, and increased cumulative productivity demonstrates that HS3+E significantly enhances filtration efficiency. The regime-based modeling approach further reveals that these improvements are not limited to a single stage but extend across all phases of the filtration process, highlighting the importance of controlling fouling mechanisms throughout operation.

### 3.2. Particle Size Distribution

**Figure 3:**
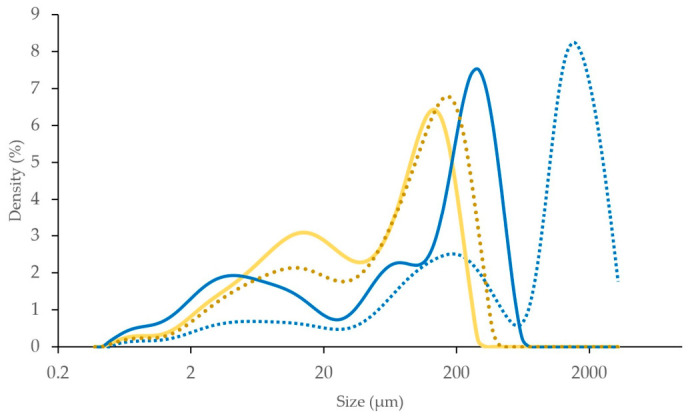
Particle size distribution of Andean blackberry hydropressed juice. GR (blue point line): milling; GR+E: milling with enzymatic treatment (blue compact line); HS3 (yellow point line): milling followed by homogenization using a three-stage rotor–stator system; HS3+E (continue yellow line): milling and homogenization with a three-stage rotor–stator system combined with enzymatic treatment.

**Figure A5:**
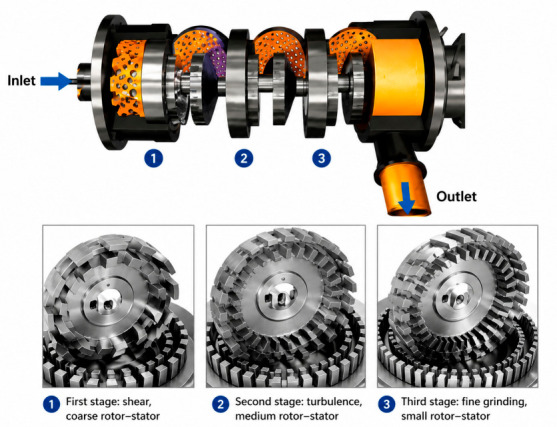
Three-stage rotor–stator tri-emulsifying pump (TRL-L3-60, Wenzhou Rayen Machinery Co., Ltd., China), powered by a 4.0-kW Siemens motor operating at 2900 rpm.

The CFM process included size reduction using a GR, followed by an HS3, and subsequently an enzymatic maceration step. To evaluate the effect of HS3, the particle size distribution was analyzed in hydropressed samples before and after enzymatic maceration. Results show (Figure 3 and Table 2) the GR with two peaks with the biggest high size particles, while the HS3 treatment was performed at a flow rate of 1.5 m^3^/h and 2900 rpm for 7 min. Considering a 30-kg batch and assuming a density close to 1 kg/L, the mixture passed through the rotor–stator system approximately six times. This repeated recirculation through the three-stage teeth configuration (coarse, middle, and fine, see Appendix F, Figure A5) promoted progressive particle disruption. The coarse stage favored initial tissue breakdown, the middle stage enhanced particle dispersion and refinement, and the fine stage contributed to further homogenization. As a result, the particle size distribution shifted toward smaller and more uniform particles, as observed in the solid blue curve in Figure 3. Therefore, the particles sizes were highly reduced for the HS3 effect. After enzymatic maceration, the impact was evident on the particle size distribution, especially for the GR treatment (dotted blue line, Figure 3). For the HS3 treatment, the enzymatic maceration reduces the particle size for the main peak size from 170 µm to 135 µm and increased the particles of the minor size until 44% (see Figure 3 and Table 2).

To facilitate comparison with the published literature, the characteristic particle size parameters Dx(10), Dx(50), and Dx(90) are presented in Appendix G, Table A5. Consistent with the peak distribution analysis, HS3 homogenization markedly reduced particle size throughout the distribution range. The median particle size, Dx(50), decreased from 977.33 ± 89.67 µm for the GR treatment to 81.53 ± 1.50 µm after HS3 processing, while Dx(90) decreased from 2300.00 ± 30.00 µm to 229.33 ± 1.53 µm, indicating a substantial reduction in the coarse particle fraction. The combination of HS3 homogenization and enzymatic treatment (HS3+E) further reduced Dx(50) and Dx(90) to 47.13 ± 4.80 µm and 172.33 ± 4.51 µm, respectively, confirming the synergistic effect of mechanical disruption and enzymatic hydrolysis on particle size reduction. These results agree with the trends observed in Figure 3 and Table 2 and demonstrate that the combined treatment produced the most homogeneous particle size distribution.

These structural modifications had a direct impact on filtration performance. From a mechanistic perspective, improved filtration performance can be explained by the changes in particle size distribution and structure induced by the HS3 treatment after the enzymatic hydrolysis. The presence of higher (290 µm main peak) particles in the GR-treated sample is known to promote pore blocking and internal fouling, leading to rapid flux decline and increased hydraulic resistance. In contrast, the reduction in fines and the formation of larger or aggregated particles (135 µm main peak) in the HS3+E treated sample favor the formation of a more porous and permeable cake layer, which reduces specific cake resistance and enhances flux stability [15]. Additionally, the application of high shear before enzymatic treatment likely enhances enzyme accessibility by increasing cell disruption and exposing intracellular components, including pectin. This can lead to more effective depectination, reducing viscosity and modifying inter-particle interactions and adhesivity to membrane material [36,37]. Lower viscosity and reduced pectin content are known to improve permeate flux in fruit juice microfiltration by decreasing resistance to flow and limiting the formation of dense fouling layers.

The Hermia analysis supports this interpretation, indicating that cake filtration is the dominant fouling mechanism for both treatments (R^2^ = 0.82–0.99, see Appendix H, Table A6). However, despite the similarity in the governing mechanism, the significantly higher flux observed for HS3+E demonstrates that the structure and permeability of the cake layer differ substantially between treatments. This confirms that fouling behavior is not only determined by the dominant mechanism, but also by the physicochemical properties of the deposited layer.

These findings are consistent with previous studies. Song et al. [33] described flux decline in crossflow microfiltration as a transition toward a cake-dominated regime, where the properties of the cake layer govern filtration performance. Similarly, Bacchin [38] highlighted the critical role of particle size and interactions in determining cake structure and permeability. In food systems, several authors have reported that reducing the fraction of fine particles and modifying pectin content significantly improve filtration performance by promoting the formation of less compact and more permeable fouling layers [39].

### 3.3. Effect of Processing Volume on Flux Performance of HS3+E-Treated Samples

**Figure 4:**
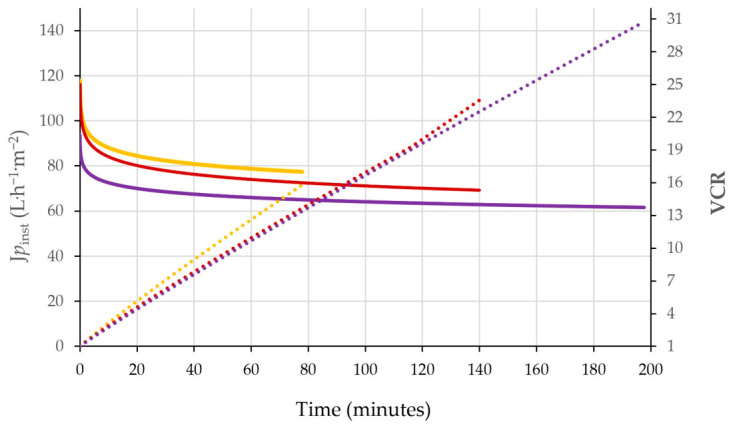
Effect of processing volumes HS-3+E on *J_px_* during crossflow microfiltration of HS3+E-treated samples. Solid lines represent permeate flux (L∙h^−1^∙m^−2^), and dashed lines represent VCR. Line colors: Yellow (200 L), Red (300 L), Purple (400 L). The secondary axis represents the VCR achieved over time. The colors with points used for the VCR profiles correspond to the same used for the *J_px_* curves on the primary axis.

Figure 4 shows the evolution of *J_px_* for HS3+E-treated samples at processing volumes of 200, 300, and 400 L. In all cases, a similar flux decline pattern was observed, characterized by an initial rapid decrease followed by a gradual stabilization, indicating that the fundamental fouling behavior remains consistent across scales. However, clear differences in flux magnitude and decline kinetics were observed as a function of processing volume.

The HS3+E-200-L condition exhibited the highest initial J_px_ (L·h^−1^·m^−2^), followed by HS3+E-300 L (93.1 L·h^−1^·m^−2^) and HS3+E-400 L (83.3 L·h^−1^·m^−2^), suggesting that an intermediate processing volume provides optimal conditions for feed structuring and filtration performance. Despite the lower initial flux at 400 L, all conditions converged toward similar steady-state flux values in the long-term regime 3, with average fluxes decreasing from 79.6 L·h^−1^·m^−2^ (200 L) to 63.5 L·h^−1^·m^−2^ (400 L), indicating increased resistance at larger volumes.

The regime-segmented analysis presented in Table 3 reveals that the transition times between filtration regimes increase with processing volume. Specifically, t23 increased from 21.3 min (200 L) to 50.9 min (400 L), while the duration of zone 3 expanded significantly from 56.6 min to 146.8 min. This indicates that at higher volumes, the system operates for longer periods under cake-dominated conditions, likely due to cumulative particle deposition and progressive cake growth.

The kinetic parameters further support this interpretation. The initial decay coefficient (*k*_1_) remained within the same order of magnitude (0.02–0.04 min^−1^), suggesting that early-stage fouling mechanisms are not strongly affected by the higher volume of juice processed. However, the slopes in regimes 2 and 3 (b_2_, b_3_) decreased in magnitude with increasing volume, particularly at 400 L, indicating a slower but more persistent flux decline associated with thicker and more resistant cake layers.

In terms of productivity, the cumulative permeate volume (AUC_3_ and AUC_total) increased significantly with processing volume, reaching 12,882 at 400 L compared to 6292 at 200 L. This reflects the extended filtration time and total processed volume, although it is accompanied by reduced flux efficiency. The ratio J_f_/J_0_ decreased from 0.8 (200 L) to 0.7 (300 L), indicating greater relative flux loss at intermediate scale, before slightly recovering at 400 L (0.7), suggesting a balance between fouling accumulation and hydrodynamic stabilization.

From a mechanistic perspective, the preservation of the regime 3 flux decline structure across all processed volumes confirms that fouling behavior is governed primarily by feed properties conditioned by the HS3+E treatment, rather than by the volume of juice processed. However, the shift in transition times and flux levels indicates that the higher volume of juice processed influences the extent and persistence of cake formation. Larger volumes likely promote increased particle residence time and cumulative deposition, leading to thicker and more compact fouling layers.

These findings are consistent with the classical crossflow microfiltration theory, which describes flux decline as a transition toward a cake-dominated regime controlled by particle deposition and shear-induced removal [33,38]. Previous studies have also reported that, while the dominant fouling mechanism may remain unchanged during scale-up, the structure and resistance of the cake layer can vary due to differences in solids loading and operation time. In food systems, some authors like Cassano et al. [7] and Vaillant et al. [39] have shown that extended filtration times and higher feed loads lead to increased cake thickness and reduced permeability, in agreement with the trends observed in this study.

### 3.4. Effect of HS3+E Treatment on SIS and Separation Performance

The concentration of SIS in the raw juice and retentate for GR+E and HS3+E treatments is presented in Table 4. At a feeding volume of 200 L, the HS3+E-treated sample showed a significant (*p* < 0.05) lower SIS concentration in the raw juice (1.6) compared to the GR+E-treated sample (1.96), indicating that the combined high-shear treatment and enzymatic depectination effectively modified the particulate structure and dispersion of insoluble material. Despite operating at a similar VCR (15.4), the SIS content in the retentate for HS3+E was less than 50% of that observed for the GR+E treatment. This suggests that high-shear homogenization not only reduces SIS in raw juice. In contrast, the GR+E treatment retains a higher amount of insoluble material, which has a strong negative impact on permeate flux, as previously discussed.

The concentration factor (SIS_retentate/SIS_feed) further highlights these differences. For GR+E at 200 L, the concentration factor was approximately 3.62, while for HS3+E at the same volume it was approximately 1.91. This indicates that although both treatments lead to solids accumulation in the retentate, the HS3+E system significantly reduces the SIS concentration, likely due to its effect on cohesivity, adhesivity, swelling power, etc. The lower SIS in the HS3+E feed suggests enhanced breakdown or restructuring of particulate matter during high-shear processing and enzymatic treatment.

When the feeding volume increased from 200 to 400 L under long-term CFM conditions with HS3+E treatment, the SIS concentration did not show a substantial increase (see Table 4). In the raw juice, only a slight rise was observed (from 1.65 to 1.70). For the retentate, the main increase occurred between 200 and 300 L, whereas at 400 L the change was limited to approximately 0.6 units. This is notable considering that the present long-term CFM experiments were conducted at significantly higher VCR values (23 and 30 for 300 L and 400 L, respectively).

Since the SIS in fruit juices act as primary foulants that readily contribute to membrane fouling, the reduction in SIS observed for the HS3+E treatment is relevant. This finding is consistent with previous studies on pretreatments in CFM processes, such as enzymatic depectinization, which enhances particle breakdown and reduces insoluble fractions in fruit juices. Studies by Vaillant et al. [8,10] have demonstrated that enzymatic treatments significantly improve clarification efficiency. However, the specific impact of the HS3+E treatment on SIS content, as well as its relationship with particle size distribution, have not been previously reported. Therefore, the results of this study support the hypothesis that high-shear homogenization limits particle aggregation and reduces cake layer formation, both of which are key contributors to flux decline in crossflow microfiltration systems. In this context, the HS3+E treatment appears to modify not only the amount but also the nature of the suspended solids, resulting in a fouling layer that, although present, exhibits lower resistance to permeate flow. This explains why HS3+E-treated samples maintain higher flux and productivity even at increased processing volumes.

**Figure 5:**
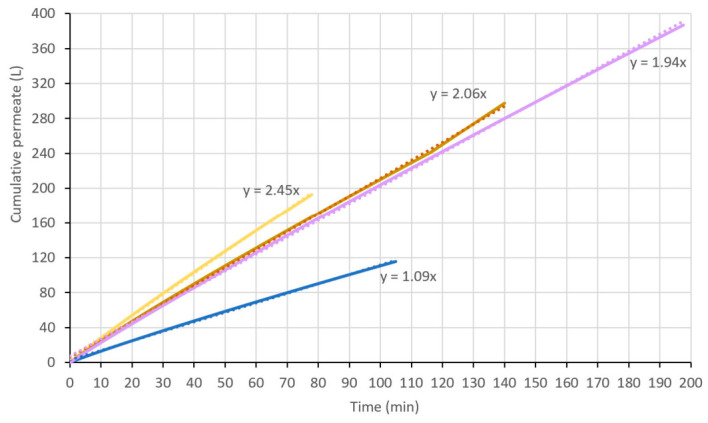
Cumulative permeates volume as a function of filtration time during the CFM process under different feeding volumes. HS3+E treatments: yellow line: 200 L, red line: 300 L, purple line: 400 L. Against the GR+E 200 L treatment blue line.

From a productivity standpoint, the reduction in particle size distribution and SIS achieved with the HS3+E treatment leads to a marked increase in cumulative processed volume during long-term CFM. As evidenced by the slopes of the regression lines in Figure 5, the GR treatment exhibits a significantly lower slope, achieving approximately 387 L of cumulative permeate over a 3-h continuous process, while other CFM studies for fruit juices typically operate within a restricted VCR range, commonly between 5 and 11 [9,40]. Our results demonstrate high-intensity concentration up to a VCR of 30. In our experiments, we observed an exponential flux decrease in the initial phase (VCR 1.8 to 2.2), likely due to the rapid development of a concentration polarization layer, followed by a stabilized region that sustained productivity until the termination of the 180-min run. This performance contrasts with traditional studies where permeate flux typically suffers from severe, continuous decline as concentration ratios increase [40], thereby suggesting that the HS3+E treatment modifies the fouling layer’s resistance, rendering it more permeable and amenable to high-intensity concentration.

From an industrial perspective, the maximum feasible VCR achieved during a standard workday was approximately 30 when the HS3+E treatment was applied. This limit was established considering not only the microfiltration stage but also the time required for feed preparation, enzymatic treatment, membrane CIP, and system sanitation between production cycles. Therefore, a VCR of 30 represents a realistic operational limit for continuous industrial implementation under the conditions evaluated. The electrical energy consumption and economic analysis presented later in this work were based on this operating scenario. The ability of the HS3+E treatment to maintain an average flux of approximately 65 L·h^−1^·m^−2^ at VCR values close to 30 demonstrates its potential for industrial-scale application while maintaining favorable energy efficiency and process productivity.

### 3.5. Product Quality

#### 3.5.1. Microbiology

Naturally, Andean blackberry exhibited a relatively high initial microbial load, including aerobic mesophilic bacteria, total coliforms (including fecal coliforms), lactic acid bacteria, and yeasts and molds (Table 5). These results are consistent with those reported by Horvitz et al. [41] and can be attributed to production conditions, fruit composition, and the fragile structure of blackberry tissues.

Following CFM processing, microbial counts in all permeate samples obtained from both GR+E and HS3+E treatments were below the quantification limit of the analytical method (<1.0 log CFU/mL), resulting in log reductions of 2.95 ± 0.88 and 2.27 ± 0.92 log units for aerobic mesophilic bacteria, 2.27–2.99 log units, total coliforms (including fecal coliforms) by more than 1.20–2.07 log units, lactic acid bacteria by more than 1.78–2.79 log units, and yeasts and molds by more than 4.75–5.39 log units in the GR+E and HS3+E treatments, respectively (Table 5).

The highest microbial reductions were observed for yeasts and molds in the GR+E treatment, followed by HS3+E, while coliforms in HS3+E were reduced to near the detection limit, preventing accurate quantification of the actual reduction magnitude. These results demonstrate the effectiveness of the CFM process combined with ultraclean packaging in substantially reducing the microbial load of Andean blackberry juice. Similar microbial removal efficiencies have been reported in previous studies on fruit juice microfiltration [42,43].

#### 3.5.2. Bioactive Compound Retention

The content and retention of bioactive compounds after CFM are presented in Table 6. For both treatments, GR+E and HS3+E, permeate concentrations were generally lower than those of the raw juice, whereas retentates showed similar or higher concentrations, indicating selective accumulation of some compounds in the retained fraction. This effect was particularly evident for Sanguiin H6 (SH6), whose concentration increased markedly in the retentate, while ellagic acid (EA) showed smaller differences among streams, suggesting a greater tendency to permeate the membrane.

Bioactive compound retention was strongly affected by the upstream physical treatment applied before enzymatic depectination. In the GR+E treatment (VCR = 15.4), anthocyanins (C3G and C3R) showed moderate retention, whereas SH6 exhibited substantially higher retention than EA [9].

Similar behavior has been reported in berry juice microfiltration, where high-molecular-weight phenolics preferentially associate with suspended solids and are retained in the fouling layer [44,45]. In contrast, HS3+E significantly reduced the retention of all bioactive compounds. Anthocyanin retention decreased to approximately 4.5%, while SH6 and EA retention reached 19.5% and 5.2%, respectively (Table 6). Consequently, about 95% of anthocyanins and 80% of ellagitannins were recovered in the permeate, representing an improvement over the recovery values typically reported for conventional fruit juice microfiltration systems [9,44].

The higher retention of SH6 is likely related to its large molecular weight (≈1869 Da) and its strong affinity for suspended solids and cell wall materials. Although these molecules are much smaller than the membrane pore size, retention is mainly governed by interactions with colloidal particles and fouling deposits rather than by size exclusion. Therefore, the lower retention observed in HS3+E suggests that high-shear homogenization reduced particle-associated retention mechanisms and limited fouling layer development [46,47,48].

Previous studies have demonstrated that fouling mitigation strategies, such as backpulsing or particle size reduction, significantly decrease the retention of anthocyanins and ellagitannins during crossflow [9,49].

These findings are consistent with the higher permeate fluxes and productivity observed for HS3+E. By generating a finer and more homogeneous particle size distribution, homogenization promoted a less resistant fouling layer, improving mass transfer and enhancing the recovery of anthocyanins and ellagitannins [45,49]. Overall, the results demonstrate that pretreatment plays a critical role in controlling bioactive compound partitioning during CFM and that high-shear homogenization can improve both process performance and product quality under high volumetric concentration ratio conditions (VCR > 30), supporting the industrial feasibility of the process.

#### 3.5.3. Sensory Profile of Microfiltered Andean Blackberry Juice

**Figure 6:**
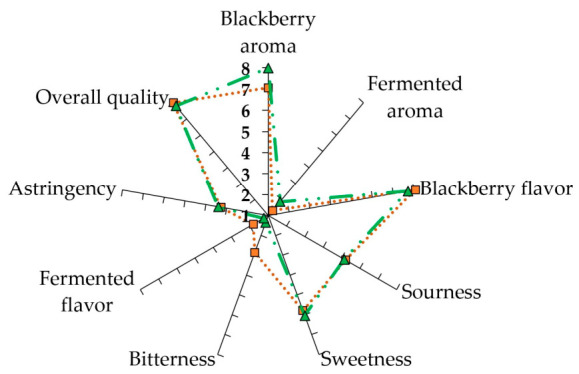
Sensory attribute profiles of microfiltered Andean blackberry juices produced using GR+E (orange squares) and HS3+E (green triangles) treatments.

The sensory profiles of microfiltered Andean blackberry juice produced from the GR+E and HS3+E treatments are presented in Figure 6. The HS3+E treatment does not adversely affect most sensory attributes of the microfiltered juice, including overall quality, blackberry flavor, sweetness, astringency, and sourness.

Notably, the HS3+E treatment significantly enhanced blackberry aroma intensity compared to the GR+E (*p* = 0.007) and reduced the bitterness (*p* < 0.0001), contributing to a more balanced sensory perception that favors blackberry aroma and taste. This suggests that the high-shear process may promote the release or preservation of volatile compounds associated with fresh fruit aroma while improving overall flavor balance. This effect can be attributed to enhanced cellular disruption, which facilitates the release of aroma precursors and volatile compounds, as reported in fruit processing studies where mechanical treatments increase aroma availability through cell wall breakdown including volatiles, thereby intensifying aroma perception [50,51]. Moreover, high-shear homogenization may liberate additional aroma precursors, such as glycosidic forms, which can be more readily cleaved by glycosidases typically present in commercial enzymatic cocktails. These findings indicate that the overall sensory balance of the juice was preserved, with improvements in aroma and taste. This is particularly relevant in an industrial context over the long term, as the results suggest that the inclusion of homogenization after milling in the CFM does not compromise the sensory quality of the product and may even contribute to a better balance of flavor and taste in the final product.

### 3.6. Economic Criteria for Blackberry Juice Production

The CMF process requires both electrical and thermal energy (see Figure 1). Thermal energy (used for sterilization and CIP procedures) was included in the cost calculations based on total plant process time. However, since the incorporation of HS3 does not affect the duration of these operations, the present analysis focuses on electrical energy consumption discussion.

#### 3.6.1. Operational and Energy Performance Under GR and HS3 Configurations

The operational performance of the GR and HS3 configurations was evaluated in terms of production capacity, specific energy consumption, and electricity cost per unit of processed beverage. As shown in Table 7, HS3 increased daily production from 187 to 387 L/day, corresponding to a 106.9% increase in throughput. This result indicates improved processability during downstream membrane operation, allowing the system to operate closer to the installed capacity.

At plant level, specific energy consumption decreased from 0.14 to 0.11 kWh L^−1^ (22.79%). Although HS3 introduced additional plant electrical demand (pump 5.4 kW and chiller 2.7 kW) associated with unit operation, the higher production throughput reduced energy intensity per unit of processed beverage. When only the CFM stage directly associated with membrane processing was considered, specific energy consumption decreased from 0.08 to 0.06 kWh L^−1^. These results confirm that HS3 improved membrane-stage energy efficiency rather than simply diluting fixed plant energy loads. The reduction in energy demand during HS3 + CFM operation suggests improved permeation stability, which could be associated with lower hydraulic resistance [11]. Similar behavior has been reported in membrane systems where process efficiency is strongly influenced by feed properties and fouling development during long-term operation [13,46]. From a techno-economic perspective, this distinction is relevant because membrane operating costs depend basically on permeate productivity.

The results obtained for the CFM stages in the GR+E configuration were slightly higher than those reported for blackberry juices under the same configuration by Zuluaga et al. [9] (0.04 kWh L^−1^, average flux 80 L·h^−1^·m^−2^) and Gallego et al. [43] (0.07 kWh L^−1^, average flux 60 L·h^−1^·m^−2^). Those studies were conducted under short processing times (~1 h) and low VCR values (~11), whereas the GR+E configuration in the present study operated with feed volumes approximately two-fold higher (200 L), which resulted in lower permeate flux values (33.7 L·h^−1^·m^−2^) at a VCR of 15.3, as discussed previously. The differences compared with the literature may be attributed to long-term process operation, in which flux decline becomes more pronounced over time. In addition, variations in process duration and operating conditions contribute to these discrepancies, as system behavior is inherently non-linear, making it difficult to extrapolate long-term performance from short-term experiments. Interestingly, when HS3 was applied, the average flux increased to 65 L·h^−1^·m^−2^ even at a higher VCR of 30. As a result, the specific energy consumption decreased significantly and reached values close to those reported in the other studies [9,43]. These findings demonstrate that the impact of homogenization is not limited to flux stabilization, but also that the process was ~22% more energy-efficient than the conventional particle size reduction process.

#### 3.6.2. System-Level and Economic Implications

A detailed economic assessment was conducted considering operating expenditures including raw materials, process inputs, cleaning agents, filtration elements, and maintenance of key equipment (boiler, compressor, refrigeration unit, packaging system, and core processing line). Economic performance was evaluated using standard financial indicators NPV, IRR, payback period, and valuation multiples (EV/Sales and EV/EBITDA) to assess profitability and investment feasibility under different process configurations [52].

At system level (see Table 8), HS3 increased production (107%) capacity from 4488 to 9288 L/month without modifications to plant infrastructure, indicating that process limitations under GR conditions were primarily operational rather than capital-dependent.

Blackberry beverage production cost decreased from 3.92 to 2.84 USD/L (27.55%) reflecting improved fixed-cost absorption and economies of scale. Although total OPEX increased (+64.6%), this rise is primarily driven by production scale rather than reduced efficiency, including higher raw material consumption, packaging, transportation, utility operation, and additional labor requirements. The HS3 configuration required five operators to support processing and handling activities, just one more worker than GR, contributing to the increase in labor-related operating costs. Despite this increase in OPEX, monthly profit increased from 5282 to 7910 USD/month (+49.76%), reflecting improved utilization of installed capacity. Economic feasibility indicators further supported the HS3 configuration. GR+E generated a negative NPV (−2364 USD) and no calculable IRR, whereas HS3+E produced a positive NPV (252 USD), an IRR of 21%, and a payback period of 60 months. These improvements were achieved with only a 4.15% increase in machinery and equipment CAPEX, which related the cost of the HS3 equipment, while infrastructure investment remained unchanged.

Overall, the economic advantage of HS3 was primarily associated with increased throughput and improved membrane-stage efficiency rather than reductions in absolute plant electrical energy consumption. Overall, the results indicate that scale and capacity utilization are the dominant drivers of economic performance. These findings are consistent with previous studies highlighting energy and operating costs as key determinants of economic feasibility in juice processing systems [29,53].

## 4. Conclusions

This study demonstrated that the productivity and techno-economic feasibility of CFM for Andean blackberry juice are strongly influenced by fouling dynamics associated with feed physicochemical characteristics generated during upstream processing. The conventional grinder treatment produced higher particle size distribution and suspended insoluble solids, leading to rapid and largely irreversible flux decline. In contrast, the incorporation of a high shear homogenization prior to enzymatic depectination significantly reduced particle size and SIS content, resulting in higher initial permeate flux, improved flux stability, and greater cumulative productivity during long-term operation, even at high volumetric reduction factors (VCR ≈ 30) and processing volumes up to 400 L. The regime-based flux analysis indicated that high-shear homogenization prior to enzymatic depectination enhanced process stability, sustaining higher permeate fluxes during long-term operation. Importantly, HS3+E did not compromise product quality, ensuring microbial reduction while improving the recovery of anthocyanins and ellagitannins in the permeate. Sensory quality was also enhanced through reduced bitterness and increased Andean blackberry aroma. From a techno-economic perspective, HS3+E more than doubled production capacity, reduced beverage production cost and energy consumption, and achieved positive economic indicators, including a positive NPV, a 21% IRR, and a 60-month payback period, while the conventional GR+E configuration remained economically unfeasible. Overall, the results demonstrate that high-shear homogenization prior to enzymatic depectination is an effective strategy to improve process productivity, product quality, energy efficiency, and industrial feasibility of long-term CFM processing for small- and medium-scale agro-industrial applications.

Since the experiments were conducted under workday production cycles, further studies are required to evaluate long-term membrane service life under HS3 pretreatment conditions over extended periods of industrial operation. In addition, the applicability of the HS3 pretreatment should be assessed in other berry fruits and raw materials processed by crossflow microfiltration to determine its potential for particle size reduction and flux stabilization in different industrial settings. Because the HS3 treatment resulted in lower retention of bioactive compounds in the retentate, future studies should also evaluate the functional properties of the resulting beverages and explore strategies for the valorization of by-products generated during processing.

## Figures and Tables

**Table 1:** Flux decline modeling parameters and performance metrics for GR+E and HS3+E treatments across filtration regimes.

Regime	1	2	3
Time GR+E (min)	0–4.70	4.70–41.45	441.45–179.95
Time HS3+E (min)	0–2.28	2.28–18.70	18.70–77.95
Model GR+E	J = 48.33e ^−0.046t^	J = 39.57 − 0.157t	J = 34.30 − 0.03t
Model HS3+E	J = 94.25e ^−0.038t^	J = 87.05 − 0.296t	J = 82.49 − 0.057t
Initial Flux GR+E	55.59	40.40	33.72
Initial Flux HS3+E	98.78	87.62	82.07
Final flux GR+E	40.40	33.72	29.21
Final flux HS3	87.62	82.07	78.30
Average flux GR+E	43.45	35.94	30.84
Average flux HS3+E	90.22	83.94	78.30
AUC GR+E	288.92	1404.89	4115.54
AUC HS3+E	311.07	1488.36	4510.01

**Table 2:** Particle size (μm) distribution parameters of blackberry juice as affected by the HS3 treatment and enzymatic maceration.

Treatment	Major Peak	Density %	Min Size	Max Size	Minor Peak 2	Density %	Min Size	Max Size
GR	1526.5	56.6	624.6	2890.9	15.4	31.3	29.2	549.8
GR+E	290.4	53.8	104.6	624.6	13.6	32.5	0.8	29.2
HS3	174.3	66.9	33.1	425.9	11.9	32.1	1.1	29.2
HS3+E	135.0	54.4	37.7	329.3	13.5	44.5	1.1	33.1

**Table 3:** Regime-segmented flux decline parameters and productivity indicators for HS3+E-treated samples at different processing volumes (200–400 L).

Indicator	200	300	400
Regime transitions			
t12 (min)	3.5	4.9	6.6
t23 (min)	21.3	36.7	50.8
tmax (min)	77.9	139.9	197.6
Regime 3 (min)	56.6	103.2	146.8
Kinetic parameters			
J0	93.1	102.1	83.3
k1 (min^−1^)	0.03	0.04	0.02
B2	−0.24	−0.31	−0.15
b3	−0.06	−0.07	−0.03
Regime 3 performance			
Jstart, 3	81.3	75.8	65.9
Jend, 3	78.3	68.7	61.2
Average J3	79.6	72.3	63.5
ΔJ3	3.2	7.1	4.7
Jf/J0	0.8	0.7	0.7
Productivity			
AUC3	4507	7456	9328
AUC total	6292	10471	12882

**Table 4:** Effect of GR+E and HS3+E treatments on the distribution of suspended insoluble solids in raw juice and retentate during long-term crossflow microfiltration.

Treatment	Raw Juice Feeding Volume	Suspends Insoluble Solids in Raw Juice	VCR	Suspends Insoluble Solids in Retentate
GR+E	100	1.9 ± 0.029 a	7.7	4.1 ± 0.058 c
	200	1.9 ± 0.10 a	15.4	7.1 ± 0.032 a
HS3+E	100	1.5 ± 0.06 c	7.7	1.9 ± 0.11 e
	200	1.6 ± 0.05 bc	15.4	3.1 ± 0.10 d
	300	1.7 ± 0.03 c	23.1	4.3 ± 0.23 c
	400	1.7 ± 0.04 b	30.3	4.9 ± 0.023 b

Mean ± standard error, n = 3, different letters in each column indicate that there is a statistically significant difference (*p* < 0.05).

**Table 5:** Microbial populations and minimum log reductions achieved in permeates obtained from GR+E and HS3+E treatments.

Microbial Group	Raw Load GR+E	GR+E (log)	Raw Load HS3+E	HS3+E (log)
Aerobic mesophilic bacteria	3.99	>2.99	3.27	>2.27
Total coliforms (incl. fecal)	3.07	>2.07	2.20	>1.20
Lactic acid bacteria	2.78	>1.78	3.79	>2.79
Yeasts and molds	6.39	>5.39	5.75	>4.75

Results for microbial group are reported as log_10_ CFU/mL.

**Table 6:** Content (mg/L) and retention (%) of cyanidins and ellagitannins during CFM.

**Cyanidins (mg/L)**				
	C3G ^1^		C3R ^2^	
Sample	GR+E	HS3+E	GR+E	HS3+E
Raw juice	95.7 ± 9.7	82.3 ± 1.3	90.5 ± 18.5	89.4 ± 14.7
MF* juice	88.8 ± 9.6	76.9 ± 2.3	73.6 ± 14.0	92.5 ± 13.0
Retentate	86.3 ± 17.9	92.4 ± 4.9	94.4 ± 5.9	93.7 ± 8.2
Retention (%)	9.26 ± 2.4 a	4.5 ± 1.6 b	10.5 ± 1.1 a	4.66 ± 1.8 b
**Ellagitannins (mg/L)**				
	SH6 ^3^		EA ^4^	
Sample	GR+E	HS3+E	GR+E	HS3+E
Raw juice	36.1 ± 3.0	38.1 ± 8.0	11.9 ± 4.9	17.1 ± 3.6
MF juice	26.1 ± 12.8	24.2 ± 15.4	9.6 ± 2.0	24.1 ± 7.1
Retentate	70.5 ± 20.6	107.9 ± 25.8	7.3 ± 4.6	25.6 ± 6.6
Retention (%)	24.8 ± 6.3 a	19.5 ± 8.4 a	9.5 ± 1.8 a	5.2 ± 1.4 b

^1^ Cyanidins 3 glucoside (external standard), ^2^ C3R: Cyanidin-3-glucose equivalent C3G. ^3^ SH6: Sanguiin H6 ellagic acid equivalent, ^4^ EA: ellagic acid equivalent (external standard), MF*: microfiltered. Different letters indicate that there is a statistically significant difference (*p* < 0.05).

**Table 7:** Operational and energy performance of GR and HS3 configurations.

Metric	GR+E	HS3+E	Difference
Daily production (L day^−1^)	187	387	200
Specific energy consumption (kWh L^−1^)	0.14	0.11	0.03
CFM stages energy consumption (kWh L^−1^)	0.08	0.06	0.02

**Table 8:** Techno-economic performance of the GR and HS3 configuration of CFM process line.

Indicator	GR+E	HS3+E	Difference
* Infrastructure investment	104,036	104,036	0
* Process line equipment investment	259,992	270,803	+10,811
* OPEX (month)	9153	15,070	5917
Monthly (24 days) production in L	4488	9288	+4800
* Beverage production cost per L	3.92	2.84	−1.08
Selling cost per liter	5.10	3.69	−1.4
* Plant electricity cost per L	0.04	0.03	−0.01
* Monthly profit	5282	7910	+2628
* NPV	−2364	252	—
IRR (%)	NC	21	—
Payback period (months)	NC	60	—

* Values reported in USD; NC: Not calculable.

## Data Availability

The original contributions presented in this study are included in the article/Supplementary Material. Further inquiries can be directed to the corresponding author.

## Supplementary Materials

The following supporting information can be downloaded at: https://www.mdpi.com/article/10.3390/foods15142493/s1. Code S1: MATLAB code used for the mathematical modeling and identification of flux decline regimes during crossflow microfiltration; File S2: Experimental dataset (Excel file) containing the GR+E and HS3+E microfiltration data obtained at a feed volume of 200 L.

## Abbreviations

The following abbreviations are used in this manuscript:

Abbreviation	Meaning
BAC	Biologically active compounds
C3G	Cyanidin 3 glucoside molecule
C3R	Cyanidin 3 rutinoside molecule
CFM	Crossflow microfiltration
EA	Ellagic acid molecule
E_e_	Electrical Energy
GR	Grinder
GR+E	Grinder plus enzymatic treated juice process
HS3	High-shear homogenization using a three-stage rotor–stator tri-emulsifying pump
HS3+E	Homogenization plus enzymatic treated juice process
IRR	Internal Rate of Return
*J*p_inst_	Adjusted flux
*J_px_*	Permeate Flux (L h^−1^ m^−2^)
NPV	Net present value
SH6	Sanguiin H6
SIS	Suspended insoluble solids
TMP	Constant transmembrane pressure
VCR	Volumetric concentration ratio
η	Efficiency of electricity transmission.

## Appendix B

**Table A1:** Summary of validation parameters of 3-glucoside chloride C3G by the HPLC-PDA method.

Data	Value		
Linear range	2.5–120 mg/L		
Correlation coefficient (r^2^)	0.9995		
Limit of detection (LOD)	1.035 mg/L		
Limit of quantification (LOQ)	3.45 mg/L		
Precision			
Day	1 (n = 3)	2 (n = 3)	3 (n = 2)
Working standard solution	5.00 mg/L	50.0 mg/L	80.0 mg/L
Measurement ± RSD	5.41 mg/L ± 3.43%	47.61 mg/L ± 2.45%	78.06 mg/L ± 1.40%
Accuracy	96.84%	92.70%	

**Table A2:** Intra-day and inter-day reproducibility of assay analyzed on three different days of ellagic acid.

Data	Value	
Linear range	5–200 mg/L	
Correlation coefficient (r^2^)	0.9998	
Limit of detection (LOD)	2.08 mg/L	
Limit of quantification (LOQ)	5.093 mg/L	
Precision		
Day	1 (n = 3)	2 (n = 3)
Working standard solution	5 mg/L	80 mg/L
Measurement ± RSD	5.43 mg/L ± 1.06%	80.42 mg/L ± 0.74%
Accuracy	105.1%	

## Appendix C

**Table A3:** Economic assumptions used in the discounted cash flow analysis.

Category	Parameter	Value
Market assumptions	Product pricing method	Cost-plus pricing
	Markup over production cost	30%
Project lifetime	Operating lifetime	20 years
	Annual operating time	288 days year^−1^
Financing and taxation	Debt financing	100%
	Short-term debt amortization	1 year
	Long-term debt amortization	8 years
	Annual interest rate (Kd)	17%
	Corporate income tax rate	35%
	Discount rate	11.05%
Working capital	Accounts receivable	30 days
	Inventory period	20 days
	Accounts payable	30 days
	Other current liabilities	20 days
Depreciation	Method	Straight-line
	Buildings and civil works	20 years
	Machinery and equipment	10 years
	Office equipment	5 years

## Appendix E

**Table A4:** Segmented flux decline analysis, goodness-of-fit statistics, and uncertainty of transition times for GR+E and HS3+E treatments.

Treatment	Parameter	Estimate (min)	95% CI Lower	95% CI Upper	R^2^	RMSE
GR+E	Regime I	—	—	—	0.79	1.40
	Regime II	—	—	—	0.93	0.45
	Regime III	—	—	—	0.97	0.19
	t12	4.70	4.20	24.20	—	—
	t23	41.45	29.70	86.33	—	—
HS3+E	Regime I	—	—	—	0.79	1.16
	Regime II	—	—	—	0.94	0.36
	Regime III	—	—	—	0.97	0.15
	t12	2.28	not stable	not stable	—	—
	t23	18.70	14.45	70.16	—	—

Transition times were estimated by segmented regression of ln(λ) using minimization of total SSE across three segments. Confidence intervals were estimated by bootstrap resampling. For HS3+E t12, the bootstrap distribution was unstable and produced an interval inconsistent with the point estimate; therefore, this confidence interval was not reported.

## Appendix G

**Table A5:** Characteristic particle size distribution parameters of Andean blackberry samples subjected to grinding, high-shear homogenization, and enzymatic treatment.

Treatment	Dx(10) (μm)	Dx(50) (μm)	Dx(90) (μm)
GR	15.57 ± 3.58	977.33 ± 89.67	2300 ± 30.00
GR+E	2.97 ± 0.26	128.67 ± 25.70	371.67 ± 36.86
HS3	5.08 ± 0.13	81.53 ± 1.50	229.33 ± 1.53
HS3+E	4.42 ± 0.39	47.13 ± 4.80	172.33 ± 4.51

Values are presented as mean ± standard deviation (n = 3).

## Appendix H

**Table A6:** Coefficient of determination (R^2^) obtained from Hermia fouling models fitted to the three filtration regimes identified by segmented regression for GR+E and HS3+E treatments.

Fouling Model	Regime		
	1	2	3
GR+E			
Complete blocking	0.8	0.95	0.97
Intermediate blocking	0.84	0.96	0.98
Standard blocking	0.82	0.96	0.97
Cake filtration	0.87	0.97	0.99
Best fit mechanism	Cake filtration	Cake filtration	Cake filtration
HS3+E			
Complete blocking	0.79	0.95	0.96
Intermediate blocking	0.81	0.95	0.95
Standard blocking	0.8	0.95	0.97
Cake filtration	0.92	0.96	0.98
Best fit mechanism	Cake filtration	Cake filtration	Cake filtration

Values correspond to the linearized Hermia models fitted to each filtration regime identified by segmented regression. The fouling mechanism associated with the highest value was considered the dominant mechanism in each regime.
